# (4*E*)-4-[(2-Hy­droxy­anilino)methyl­idene]-1-phenyl­pyrazolidine-3,5-dione dimethyl sulfoxide hemisolvate

**DOI:** 10.1107/S1600536813022034

**Published:** 2013-08-10

**Authors:** Mehmet Akkurt, Shaaban K. Mohamed, Mahmoud A. A. Elremaily, Eman. A. Ahmed, Mustafa R. Albayati

**Affiliations:** a Department of Physics, Faculty of Sciences, Erciyes University, 38039 Kayseri, Turkey; b Chemistry and Environmental Division, Manchester Metropolitan University, Manchester, M1 5GD, England; c Chemistry Department, Faculty of Science, Minia University, 61519 El-Minia, Egypt; d Department of Organic Chemistry, Faculty of Science, Institute of Biotechnology, Granada University, Granada E-18071, Spain; e Department of Chemistry, Sohag University, 82524 Sohag, Egypt; f Department of Chemistry, University of Leicester, Leicester, England; g Kirkuk University, College of Science, Department of Chemistry, Kirkuk, Iraq

## Abstract

The asymmetric unit of the title compound, C_16_H_13_N_3_O_3_·0.5C_2_H_6_OS, is composed of two independent pyrazolidine-3,5-dione mol­ecules and one dimethyl sulfoxide solvent mol­ecule. In each pyrazolidine-3,5-dione mol­ecule, an intra­molecular N—H⋯O hydrogen bond forms an *S*(5)*S*(6) motif. In the crystal, pairs of each independent pyrazolidine-3,5-dione mol­ecule are linked by N—H⋯O hydrogen bonds, forming dimers with *R*_2_^2^(8) motifs. These dimers are connected with the other mol­ecules through the solvent mol­ecules *via* O—H⋯O hydrogen bonds, forming ribbons along the *b*-axis direction. C—H⋯π inter­actions connect the ribbons. C—H⋯O interactions also occur.

## Related literature

For the significant role of pyrazolidinediones in the synthesis of various heterocyclic compounds, see: Elnagdy & Ohta (1973 ▶); Abdel-Rahman *et al.* (2004 ▶); Khodairy (2007 ▶). For the diverse biological actvities of pyrazolidinedione-containing compounds, see: D’Alo *et al.* (1978 ▶); Tawab *et al.* (1960 ▶). For graph-set motifs, see: Bernstein *et al.* (1995 ▶).
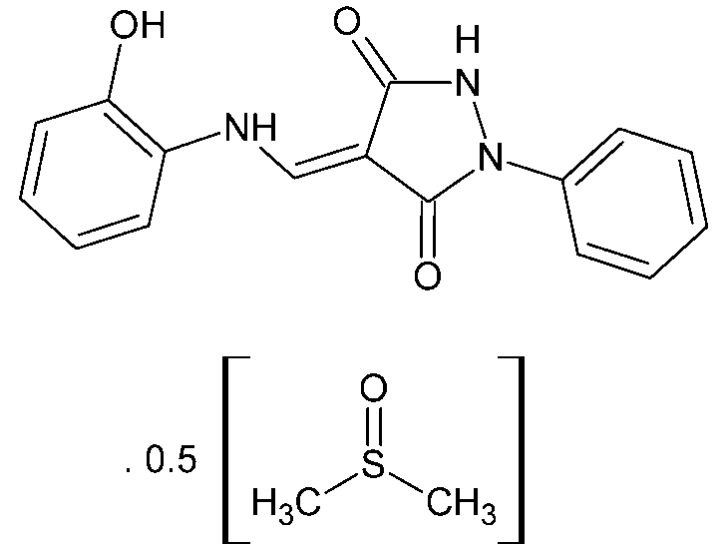


## Experimental

### 

#### Crystal data

C_16_H_13_N_3_O_3_·0.5C_2_H_6_OS
*M* *_r_* = 334.37
Triclinic, 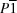

*a* = 5.7740 (2) Å
*b* = 14.9402 (6) Å
*c* = 19.2441 (7) Å
α = 106.060 (1)°
β = 93.459 (1)°
γ = 92.653 (1)°
*V* = 1588.96 (10) Å^3^
*Z* = 4
Mo *K*α radiation
μ = 0.16 mm^−1^
*T* = 100 K
0.47 × 0.14 × 0.06 mm

#### Data collection

Bruker APEXII CCD diffractometer
Absorption correction: multi-scan (*SADABS*; Bruker, 2012 ▶) *T*_min_ = 0.973, *T*_max_ = 0.990
25749 measured reflections
7370 independent reflections
5874 reflections with *I* > 2σ(*I*)
*R*_int_ = 0.043

#### Refinement

*R*[*F* ^2^ > 2σ(*F* ^2^)] = 0.044
*wR*(*F* ^2^) = 0.112
*S* = 1.02
7370 reflections
453 parameters
6 restraints
H-atom parameters constrained
Δρ_max_ = 0.40 e Å^−3^
Δρ_min_ = −0.51 e Å^−3^

### 

Data collection: *APEX2* (Bruker, 2013 ▶); cell refinement: *SAINT* (Bruker, 2013 ▶); data reduction: *SAINT*; program(s) used to solve structure: *SIR97* (Altomare *et al.*, 1999 ▶); program(s) used to refine structure: *SHELXL97* (Sheldrick, 2008 ▶); molecular graphics: *ORTEP-3 for Windows* (Farrugia, 2012 ▶); software used to prepare material for publication: *WinGX* (Farrugia, 2012 ▶) and *PLATON* (Spek, 2009 ▶).

## Supplementary Material

Crystal structure: contains datablock(s) global, I. DOI: 10.1107/S1600536813022034/hg5338sup1.cif

Structure factors: contains datablock(s) I. DOI: 10.1107/S1600536813022034/hg5338Isup2.hkl

Supplementary material file. DOI: 10.1107/S1600536813022034/hg5338Isup3.cml

Additional supplementary materials:  crystallographic information; 3D view; checkCIF report

## Figures and Tables

**Table 1:** Hydrogen-bond geometry (Å, °) *Cg*3 and *Cg*6 are the centroids of the C11–C16 and C27–C32 phenyl rings, respectively.

*D*—H⋯*A*	*D*—H	H⋯*A*	*D*⋯*A*	*D*—H⋯*A*
N1—H1⋯O3	0.86 (2)	2.15 (2)	2.8265 (17)	135 (2)
O1—H1*A*⋯O5	0.85 (2)	1.80 (2)	2.6479 (17)	174 (2)
N3—H3*A*⋯O2^i^	0.88 (2)	1.90 (2)	2.7740 (17)	174 (2)
N4—H4*A*⋯O6	0.88 (2)	2.11 (2)	2.8050 (18)	136 (2)
O4—H4*B*⋯O7^ii^	0.85 (2)	1.76 (2)	2.6061 (18)	172 (2)
N6—H6⋯O6^iii^	0.88 (2)	1.92 (2)	2.7831 (19)	169 (2)
C34—H34*B*⋯O3^iv^	0.98	2.43	3.403 (3)	175
C29—H29⋯*Cg*3^v^	0.95	2.64	3.548 (2)	160
C33—H33*C*⋯*Cg*6^vi^	0.98	2.74	3.690 (2)	163

Symmetry codes: (i) 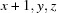; (ii) 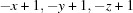; (iii) 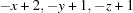; (iv) 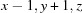; (v) 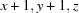; (vi) 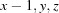.

**Fig. 1.**
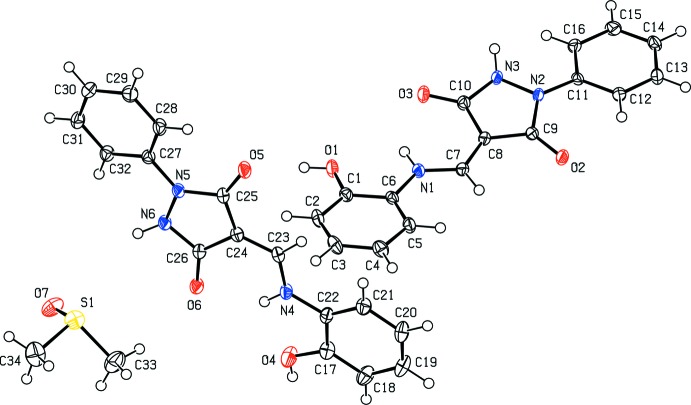
The molecular structure of the title compound showing the atom numbering scheme. Displacement ellipsoids for non-H atoms are drawn at the 50% probability level.

**Fig. 2.**
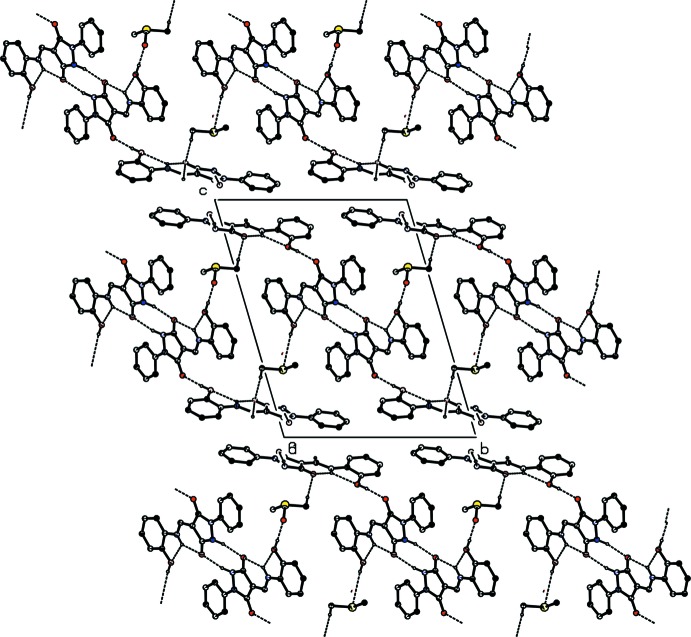
View of the N—H···O dimers and O—H···O and N—H···O hydrogen bonding (dashed lines) down the b axis of the title compound.

## supplementary crystallographic information

### 1. Comment

Pyrazolidinedione compounds have been used as a core precursor to prepare a diversity of heterocyclic systems owning to the acidic methylene function (Khodairy, 2007; Abdel-Rahman *et al.*, 2004); Elnagdy & Ohta, 1973). Moreover, pyrazolidinones have exhibited a wide spectrum of biological activities such as antipyretic (Tawab *et al.*, 1960) and antiinflammatory drugs (D'Alo *et al.*, 1978). In this concept, we herein report the synthesis and crystal structure of the title compound.

As shown in Fig. 1, the asymmetric unit of the title compound (I) contains two crystallographically independent molecules (A with O1 and B with O4) of (4E)-4-{[(2-hydroxyphenyl)amino]methylidene}-1-phenylpyrazolidine-3,5-dione and one molecule of dimethyl sulfoxide solvate. In molecule A, the benzene and phenyl rings are oriented at dihedral angles of 15.87 (8) and 9.97 (8) ° with respect to the pyrazolidine ring. In molecule B, the corresponding angles are 6.55 (9) and 9.80 (9) °, respectively.

Intramolecular N—H···O hydrogen bonds form S(5)S(6) motifs (Bernstein *et al.*, 1995). Pairs of molecules are linked by N—H···O forming a dimer with *R*^2^_2_(8) motifs (Table 1, Fig. 2). These dimers are also connected with the other molecules through the DMSO solvate molecules *via* O—H···O hydrogen generating bonds ribbons along b a-xis. Furthermore C—H···π interactions are observed between the ribbons (Table 1).

### 2. Experimental

A mixture of (4*Z*)-4-[(dimethylamino)methylidene]-1-phenylpyrazolidine-3,5-dione (231 mg, 1 mmol) and 2-aminophenol (109 mg, 1 mmol) in 50 ml acetic acid was refluxed for 2 h. The resulting solid on hot was filtered off, dried under vacuum, washed with ethanol and recrystallized from dimethyl sufoxide to afford the title compound in good quality crystals (*M*.p.: 541 - 542 K) sufficient for X-ray diffraction.

### 3. Refinement

The C-bound H atoms were placed in geometrically idealized positions [C—H = 0.95 Å for aromatic H and C—H = 0.98 Å for methyl H] and were refined using a riding model with *U*_iso_(H) = 1.2*U*_eq_(C_aromatic_) and 1.5*U*_eq_(C_methyl_). Hydroxyl and amine H atoms are found from difference Fourier maps and refined with constraints of N—H = 0.88 (2) and O—H = 0.84 (2) Å, *U*_iso_(H) = 1.2*U*_eq_(N) for amine H atoms and *U*_iso_(H) = 1.5*U*_eq_(O) for hydroxyl H atoms.

### Crystal data

**Table:**

C_16_H_13_N_3_O_3_·0.5C_2_H_6_OS	*Z* = 4
*M**_r_* = 334.37	*F*(000) = 700
Triclinic, *P*1	*D*_x_ = 1.398 Mg m^−^^3^
Hall symbol: -P 1	Mo *K*α radiation, λ = 0.71073 Å
*a* = 5.7740 (2) Å	Cell parameters from 9951 reflections
*b* = 14.9402 (6) Å	θ = 2.8–27.6°
*c* = 19.2441 (7) Å	µ = 0.16 mm^−^^1^
α = 106.060 (1)°	*T* = 100 K
β = 93.459 (1)°	Prism, colourless
γ = 92.653 (1)°	0.47 × 0.14 × 0.06 mm
*V* = 1588.96 (10) Å^3^	

### Data collection

**Table:**

Bruker APEXII CCD diffractometer	7370 independent reflections
Radiation source: sealed tube	5874 reflections with *I* > 2σ(*I*)
Graphite monochromator	*R*_int_ = 0.043
φ and ω scans	θ_max_ = 27.6°, θ_min_ = 2.3°
Absorption correction: multi-scan (*SADABS*; Bruker, 2012)	*h* = −7→6
*T*_min_ = 0.973, *T*_max_ = 0.990	*k* = −19→19
25749 measured reflections	*l* = −24→25

### Refinement

**Table:**

Refinement on *F*^2^	6 restraints
Least-squares matrix: full	H-atom parameters constrained
*R*[*F*^2^ > 2σ(*F*^2^)] = 0.044	W = 1/[Σ^2^(*F*O^2^) + (0.0457*P*)^2^ + 1.0131*P*] WHERE *P* = (*F*O^2^ + 2*F*C^2^)/3
*wR*(*F*^2^) = 0.112	(Δ/σ)_max_ = 0.001
*S* = 1.02	Δρ_max_ = 0.40 e Å^−^^3^
7370 reflections	Δρ_min_ = −0.51 e Å^−^^3^
453 parameters	

### Special details

**Table:**

Geometry. Bond distances, angles *etc*. have been calculated using the rounded fractional coordinates. All su's are estimated from the variances of the (full) variance-covariance matrix. The cell e.s.d.'s are taken into account in the estimation of distances, angles and torsion angles
Refinement. Refinement on *F*^2^ for ALL reflections except those flagged by the user for potential systematic errors. Weighted *R*-factors *wR* and all goodnesses of fit *S* are based on *F*^2^, conventional *R*-factors *R* are based on *F*, with *F* set to zero for negative *F*^2^. The observed criterion of *F*^2^ > σ(*F*^2^) is used only for calculating -*R*-factor-obs *etc*. and is not relevant to the choice of reflections for refinement. *R*-factors based on *F*^2^ are statistically about twice as large as those based on *F*, and *R*-factors based on ALL data will be even larger.

### Fractional atomic coordinates and isotropic or equivalent isotropic displacement parameters (Å^2^)

**Table:**

	*x*	*y*	*z*	*U*_iso_*/*U*_eq_	
O1	1.0470 (2)	0.31031 (8)	0.80151 (7)	0.0235 (3)	
O2	0.65665 (19)	−0.04836 (8)	0.93836 (7)	0.0239 (4)	
O3	1.2614 (2)	0.09699 (8)	0.84559 (7)	0.0249 (4)	
N1	0.8575 (2)	0.19791 (9)	0.86858 (7)	0.0176 (4)	
N2	1.0350 (2)	−0.07965 (9)	0.91086 (7)	0.0165 (4)	
N3	1.2123 (2)	−0.04078 (9)	0.87775 (8)	0.0187 (4)	
C1	0.8647 (3)	0.34333 (11)	0.83966 (8)	0.0182 (4)	
C2	0.7821 (3)	0.43089 (11)	0.84613 (9)	0.0236 (5)	
C3	0.5912 (3)	0.45743 (11)	0.88502 (10)	0.0258 (5)	
C4	0.4791 (3)	0.39733 (11)	0.91716 (9)	0.0236 (5)	
C5	0.5609 (3)	0.30982 (11)	0.91163 (9)	0.0203 (4)	
C6	0.7542 (3)	0.28328 (10)	0.87382 (8)	0.0166 (4)	
C7	0.7803 (3)	0.12843 (10)	0.89313 (8)	0.0174 (4)	
C8	0.9069 (3)	0.05182 (10)	0.89051 (8)	0.0162 (4)	
C9	0.8422 (3)	−0.02722 (10)	0.91639 (8)	0.0162 (4)	
C10	1.1384 (3)	0.04256 (10)	0.86849 (9)	0.0178 (4)	
C11	1.0542 (3)	−0.17105 (10)	0.91722 (8)	0.0154 (4)	
C12	0.8869 (3)	−0.21227 (11)	0.95024 (8)	0.0184 (4)	
C13	0.9161 (3)	−0.30103 (11)	0.95758 (9)	0.0217 (5)	
C14	1.1073 (3)	−0.34943 (11)	0.93344 (9)	0.0228 (5)	
C15	1.2715 (3)	−0.30817 (11)	0.90057 (10)	0.0238 (5)	
C16	1.2464 (3)	−0.21971 (11)	0.89186 (9)	0.0200 (5)	
O4	0.3509 (2)	0.22169 (9)	0.47285 (7)	0.0331 (4)	
O5	1.2172 (2)	0.43434 (8)	0.73963 (6)	0.0242 (4)	
O6	0.8410 (2)	0.39366 (8)	0.50665 (6)	0.0230 (3)	
N4	0.6176 (2)	0.29482 (9)	0.59083 (7)	0.0197 (4)	
N5	1.2770 (2)	0.49105 (9)	0.64119 (7)	0.0179 (4)	
N6	1.1791 (2)	0.47253 (10)	0.56906 (7)	0.0195 (4)	
C17	0.2873 (3)	0.19598 (12)	0.53154 (9)	0.0244 (5)	
C18	0.0972 (3)	0.13555 (13)	0.53120 (10)	0.0299 (5)	
C19	0.0470 (3)	0.11388 (13)	0.59430 (11)	0.0321 (6)	
C20	0.1841 (3)	0.15263 (13)	0.65813 (10)	0.0305 (6)	
C21	0.3754 (3)	0.21364 (12)	0.65940 (9)	0.0248 (5)	
C22	0.4276 (3)	0.23438 (11)	0.59601 (9)	0.0206 (5)	
C23	0.7898 (3)	0.33169 (11)	0.64012 (9)	0.0188 (4)	
C24	0.9671 (3)	0.38897 (11)	0.62757 (8)	0.0181 (4)	
C25	1.1586 (3)	0.43738 (10)	0.67724 (8)	0.0182 (4)	
C26	0.9804 (3)	0.41578 (11)	0.56190 (8)	0.0186 (4)	
C27	1.4860 (3)	0.54793 (10)	0.66204 (8)	0.0176 (4)	
C28	1.5761 (3)	0.57334 (12)	0.73415 (9)	0.0228 (5)	
C29	1.7817 (3)	0.62903 (12)	0.75405 (10)	0.0270 (5)	
C30	1.8980 (3)	0.66043 (12)	0.70351 (10)	0.0250 (5)	
C31	1.8058 (3)	0.63606 (12)	0.63225 (9)	0.0232 (5)	
C32	1.6008 (3)	0.58009 (11)	0.61100 (9)	0.0206 (5)	
S1	0.71959 (8)	0.90279 (3)	0.71557 (2)	0.0305 (2)	
O7	0.8621 (2)	0.87145 (9)	0.65113 (7)	0.0334 (4)	
C33	0.4512 (4)	0.83276 (15)	0.69636 (13)	0.0416 (7)	
C34	0.6075 (4)	1.00954 (15)	0.70907 (12)	0.0418 (7)	
H1	0.990 (3)	0.1921 (13)	0.8499 (10)	0.0210*	
H1A	1.108 (4)	0.3515 (13)	0.7841 (11)	0.0350*	
H2	0.85680	0.47250	0.82380	0.0280*	
H3	0.53640	0.51760	0.88970	0.0310*	
H3A	1.352 (3)	−0.0388 (13)	0.8987 (10)	0.0220*	
H4	0.34620	0.41580	0.94300	0.0280*	
H5	0.48430	0.26840	0.93370	0.0240*	
H7	0.63340	0.13150	0.91310	0.0210*	
H12	0.75470	−0.17990	0.96750	0.0220*	
H13	0.80170	−0.32920	0.97980	0.0260*	
H14	1.12570	−0.40990	0.93930	0.0270*	
H15	1.40370	−0.34080	0.88370	0.0290*	
H16	1.35980	−0.19250	0.86870	0.0240*	
H4A	0.627 (3)	0.3061 (13)	0.5487 (9)	0.0240*	
H4B	0.269 (4)	0.1914 (16)	0.4344 (10)	0.0500*	
H6	1.177 (3)	0.5205 (11)	0.5509 (10)	0.0230*	
H18	0.00160	0.10910	0.48770	0.0360*	
H19	−0.08260	0.07210	0.59380	0.0390*	
H20	0.14760	0.13760	0.70120	0.0370*	
H21	0.46890	0.24070	0.70320	0.0300*	
H23	0.79180	0.31840	0.68570	0.0230*	
H28	1.49740	0.55270	0.76940	0.0270*	
H29	1.84370	0.64590	0.80310	0.0320*	
H30	2.03920	0.69820	0.71760	0.0300*	
H31	1.88370	0.65790	0.59740	0.0280*	
H32	1.53910	0.56380	0.56190	0.0250*	
H33A	0.38080	0.83310	0.64880	0.0620*	
H33B	0.34500	0.85820	0.73370	0.0620*	
H33C	0.48070	0.76860	0.69600	0.0620*	
H34A	0.73610	1.05680	0.71500	0.0630*	
H34B	0.50090	1.03090	0.74710	0.0630*	
H34C	0.52370	0.99960	0.66140	0.0630*	

### Atomic displacement parameters (Å^2^)

**Table:**

	*U*^11^	*U*^22^	*U*^33^	*U*^12^	*U*^13^	*U*^23^
O1	0.0266 (6)	0.0209 (6)	0.0281 (6)	0.0018 (5)	0.0080 (5)	0.0139 (5)
O2	0.0160 (6)	0.0201 (6)	0.0412 (7)	0.0035 (4)	0.0091 (5)	0.0161 (5)
O3	0.0213 (6)	0.0220 (6)	0.0378 (7)	0.0022 (5)	0.0096 (5)	0.0174 (5)
N1	0.0173 (7)	0.0149 (6)	0.0222 (7)	0.0021 (5)	0.0031 (5)	0.0075 (5)
N2	0.0139 (6)	0.0140 (6)	0.0243 (7)	0.0008 (5)	0.0050 (5)	0.0090 (5)
N3	0.0135 (6)	0.0177 (6)	0.0290 (7)	0.0021 (5)	0.0062 (6)	0.0124 (6)
C1	0.0208 (8)	0.0168 (7)	0.0170 (8)	−0.0007 (6)	−0.0019 (6)	0.0059 (6)
C2	0.0320 (9)	0.0171 (8)	0.0248 (9)	0.0001 (7)	0.0017 (7)	0.0115 (6)
C3	0.0376 (10)	0.0145 (8)	0.0274 (9)	0.0065 (7)	0.0013 (7)	0.0088 (6)
C4	0.0277 (9)	0.0195 (8)	0.0246 (9)	0.0073 (7)	0.0046 (7)	0.0065 (6)
C5	0.0233 (8)	0.0163 (7)	0.0236 (8)	0.0017 (6)	0.0031 (7)	0.0091 (6)
C6	0.0198 (8)	0.0121 (7)	0.0175 (8)	0.0003 (6)	−0.0029 (6)	0.0047 (6)
C7	0.0159 (7)	0.0147 (7)	0.0217 (8)	0.0001 (6)	0.0003 (6)	0.0058 (6)
C8	0.0158 (7)	0.0141 (7)	0.0190 (8)	−0.0011 (6)	0.0010 (6)	0.0056 (6)
C9	0.0152 (7)	0.0141 (7)	0.0200 (8)	0.0015 (6)	0.0014 (6)	0.0057 (6)
C10	0.0185 (8)	0.0148 (7)	0.0209 (8)	0.0015 (6)	0.0028 (6)	0.0061 (6)
C11	0.0168 (7)	0.0119 (7)	0.0176 (7)	−0.0001 (6)	−0.0024 (6)	0.0052 (5)
C12	0.0179 (8)	0.0183 (7)	0.0202 (8)	0.0018 (6)	0.0026 (6)	0.0073 (6)
C13	0.0241 (8)	0.0197 (8)	0.0235 (8)	−0.0024 (6)	0.0008 (7)	0.0108 (6)
C14	0.0262 (9)	0.0157 (7)	0.0283 (9)	0.0010 (6)	−0.0041 (7)	0.0103 (6)
C15	0.0194 (8)	0.0169 (8)	0.0350 (10)	0.0046 (6)	0.0004 (7)	0.0071 (7)
C16	0.0161 (8)	0.0167 (7)	0.0288 (9)	0.0007 (6)	0.0016 (6)	0.0089 (6)
O4	0.0378 (8)	0.0395 (8)	0.0194 (6)	−0.0165 (6)	−0.0023 (6)	0.0081 (5)
O5	0.0292 (7)	0.0270 (6)	0.0188 (6)	−0.0069 (5)	−0.0028 (5)	0.0126 (5)
O6	0.0244 (6)	0.0276 (6)	0.0176 (6)	−0.0066 (5)	−0.0017 (5)	0.0095 (5)
N4	0.0226 (7)	0.0205 (7)	0.0174 (7)	−0.0027 (5)	0.0025 (6)	0.0079 (5)
N5	0.0202 (7)	0.0210 (7)	0.0137 (6)	−0.0012 (5)	0.0004 (5)	0.0077 (5)
N6	0.0222 (7)	0.0236 (7)	0.0137 (6)	−0.0039 (6)	−0.0011 (5)	0.0085 (5)
C17	0.0282 (9)	0.0221 (8)	0.0219 (8)	−0.0017 (7)	0.0047 (7)	0.0043 (6)
C18	0.0293 (10)	0.0280 (9)	0.0275 (9)	−0.0079 (7)	0.0025 (7)	0.0012 (7)
C19	0.0299 (10)	0.0266 (9)	0.0373 (11)	−0.0098 (7)	0.0108 (8)	0.0048 (8)
C20	0.0343 (10)	0.0294 (9)	0.0302 (10)	−0.0033 (8)	0.0123 (8)	0.0114 (7)
C21	0.0262 (9)	0.0252 (9)	0.0237 (9)	−0.0004 (7)	0.0039 (7)	0.0079 (7)
C22	0.0204 (8)	0.0171 (8)	0.0249 (8)	−0.0006 (6)	0.0050 (7)	0.0063 (6)
C23	0.0233 (8)	0.0171 (7)	0.0178 (8)	0.0023 (6)	0.0036 (6)	0.0073 (6)
C24	0.0213 (8)	0.0171 (7)	0.0164 (8)	0.0013 (6)	0.0022 (6)	0.0056 (6)
C25	0.0214 (8)	0.0161 (7)	0.0185 (8)	0.0006 (6)	0.0035 (6)	0.0070 (6)
C26	0.0197 (8)	0.0182 (7)	0.0185 (8)	0.0004 (6)	0.0027 (6)	0.0061 (6)
C27	0.0180 (8)	0.0162 (7)	0.0191 (8)	0.0018 (6)	0.0024 (6)	0.0057 (6)
C28	0.0271 (9)	0.0242 (8)	0.0187 (8)	−0.0026 (7)	0.0022 (7)	0.0092 (6)
C29	0.0297 (10)	0.0286 (9)	0.0223 (9)	−0.0059 (7)	−0.0057 (7)	0.0095 (7)
C30	0.0206 (8)	0.0245 (8)	0.0299 (9)	−0.0047 (7)	−0.0009 (7)	0.0092 (7)
C31	0.0232 (8)	0.0244 (8)	0.0240 (9)	−0.0003 (7)	0.0069 (7)	0.0093 (7)
C32	0.0224 (8)	0.0224 (8)	0.0173 (8)	0.0000 (6)	0.0029 (6)	0.0061 (6)
S1	0.0324 (3)	0.0342 (3)	0.0215 (2)	0.0017 (2)	0.0025 (2)	0.0020 (2)
O7	0.0256 (7)	0.0396 (8)	0.0272 (7)	−0.0041 (6)	0.0038 (5)	−0.0030 (6)
C33	0.0305 (11)	0.0439 (12)	0.0501 (13)	0.0012 (9)	0.0145 (10)	0.0106 (10)
C34	0.0533 (14)	0.0372 (11)	0.0369 (11)	0.0086 (10)	0.0160 (10)	0.0102 (9)

### Geometric parameters (Å, º)

**Table:**

S1—C34	1.782 (2)	C2—H2	0.9500
S1—O7	1.5072 (13)	C3—H3	0.9500
S1—C33	1.789 (2)	C4—H4	0.9500
O1—C1	1.353 (2)	C5—H5	0.9500
O2—C9	1.234 (2)	C7—H7	0.9500
O3—C10	1.242 (2)	C12—H12	0.9500
O1—H1A	0.85 (2)	C13—H13	0.9500
O4—C17	1.355 (2)	C14—H14	0.9500
O5—C25	1.2412 (19)	C15—H15	0.9500
O6—C26	1.2511 (19)	C16—H16	0.9500
O4—H4B	0.85 (2)	C17—C22	1.402 (2)
N1—C7	1.324 (2)	C17—C18	1.387 (3)
N1—C6	1.414 (2)	C18—C19	1.384 (3)
N2—C11	1.413 (2)	C19—C20	1.385 (3)
N2—N3	1.4212 (18)	C20—C21	1.394 (3)
N2—C9	1.383 (2)	C21—C22	1.386 (2)
N3—C10	1.388 (2)	C23—C24	1.379 (2)
N1—H1	0.863 (18)	C24—C25	1.441 (2)
N3—H3A	0.875 (18)	C24—C26	1.433 (2)
N4—C23	1.318 (2)	C27—C32	1.395 (2)
N4—C22	1.414 (2)	C27—C28	1.394 (2)
N5—C27	1.413 (2)	C28—C29	1.388 (3)
N5—N6	1.4146 (18)	C29—C30	1.385 (3)
N5—C25	1.381 (2)	C30—C31	1.384 (2)
N6—C26	1.371 (2)	C31—C32	1.389 (2)
N4—H4A	0.877 (17)	C18—H18	0.9500
N6—H6	0.880 (17)	C19—H19	0.9500
C1—C6	1.403 (2)	C20—H20	0.9500
C1—C2	1.389 (2)	C21—H21	0.9500
C2—C3	1.381 (2)	C23—H23	0.9500
C3—C4	1.382 (2)	C28—H28	0.9500
C4—C5	1.389 (2)	C29—H29	0.9500
C5—C6	1.383 (2)	C30—H30	0.9500
C7—C8	1.377 (2)	C31—H31	0.9500
C8—C10	1.428 (2)	C32—H32	0.9500
C8—C9	1.445 (2)	C33—H33A	0.9800
C11—C16	1.394 (2)	C33—H33B	0.9800
C11—C12	1.397 (2)	C33—H33C	0.9800
C12—C13	1.389 (2)	C34—H34A	0.9800
C13—C14	1.381 (2)	C34—H34B	0.9800
C14—C15	1.383 (2)	C34—H34C	0.9800
C15—C16	1.390 (2)		
			
O7—S1—C33	107.82 (9)	C15—C14—H14	121.00
O7—S1—C34	105.34 (9)	C14—C15—H15	119.00
C33—S1—C34	97.86 (11)	C16—C15—H15	119.00
C1—O1—H1A	110.7 (15)	C15—C16—H16	120.00
C17—O4—H4B	111.6 (15)	C11—C16—H16	120.00
C6—N1—C7	127.16 (13)	O4—C17—C18	124.48 (16)
N3—N2—C9	110.49 (13)	O4—C17—C22	115.99 (15)
N3—N2—C11	118.54 (12)	C18—C17—C22	119.53 (16)
C9—N2—C11	129.12 (13)	C17—C18—C19	119.91 (17)
N2—N3—C10	107.55 (12)	C18—C19—C20	120.53 (17)
C7—N1—H1	116.5 (13)	C19—C20—C21	120.25 (17)
C6—N1—H1	116.2 (13)	C20—C21—C22	119.24 (16)
C10—N3—H3A	116.6 (13)	C17—C22—C21	120.52 (16)
N2—N3—H3A	114.2 (12)	N4—C22—C21	124.13 (15)
C22—N4—C23	127.91 (14)	N4—C22—C17	115.34 (15)
C25—N5—C27	130.40 (13)	N4—C23—C24	121.98 (15)
N6—N5—C25	110.15 (12)	C23—C24—C26	124.17 (15)
N6—N5—C27	118.85 (12)	C23—C24—C25	127.93 (14)
N5—N6—C26	108.04 (12)	C25—C24—C26	107.76 (14)
C22—N4—H4A	116.0 (12)	N5—C25—C24	105.83 (13)
C23—N4—H4A	116.0 (12)	O5—C25—N5	124.06 (15)
N5—N6—H6	116.1 (12)	O5—C25—C24	130.11 (15)
C26—N6—H6	119.1 (11)	N6—C26—C24	107.70 (14)
O1—C1—C2	124.32 (15)	O6—C26—C24	128.32 (16)
O1—C1—C6	116.43 (15)	O6—C26—N6	123.98 (14)
C2—C1—C6	119.25 (15)	N5—C27—C28	119.95 (14)
C1—C2—C3	119.97 (16)	C28—C27—C32	119.75 (16)
C2—C3—C4	120.71 (16)	N5—C27—C32	120.29 (14)
C3—C4—C5	119.95 (16)	C27—C28—C29	119.51 (16)
C4—C5—C6	119.72 (16)	C28—C29—C30	121.06 (17)
C1—C6—C5	120.38 (15)	C29—C30—C31	119.12 (16)
N1—C6—C1	115.82 (14)	C30—C31—C32	120.86 (16)
N1—C6—C5	123.78 (14)	C27—C32—C31	119.69 (15)
N1—C7—C8	121.87 (15)	C19—C18—H18	120.00
C7—C8—C10	124.68 (15)	C17—C18—H18	120.00
C9—C8—C10	108.23 (14)	C18—C19—H19	120.00
C7—C8—C9	126.77 (16)	C20—C19—H19	120.00
N2—C9—C8	105.69 (14)	C19—C20—H20	120.00
O2—C9—N2	124.45 (14)	C21—C20—H20	120.00
O2—C9—C8	129.85 (16)	C22—C21—H21	120.00
N3—C10—C8	107.75 (13)	C20—C21—H21	120.00
O3—C10—C8	128.75 (15)	C24—C23—H23	119.00
O3—C10—N3	123.49 (15)	N4—C23—H23	119.00
N2—C11—C16	119.35 (14)	C27—C28—H28	120.00
N2—C11—C12	121.03 (15)	C29—C28—H28	120.00
C12—C11—C16	119.60 (15)	C28—C29—H29	119.00
C11—C12—C13	119.25 (16)	C30—C29—H29	119.00
C12—C13—C14	121.64 (16)	C31—C30—H30	120.00
C13—C14—C15	118.67 (16)	C29—C30—H30	120.00
C14—C15—C16	121.10 (16)	C30—C31—H31	120.00
C11—C16—C15	119.73 (16)	C32—C31—H31	120.00
C1—C2—H2	120.00	C27—C32—H32	120.00
C3—C2—H2	120.00	C31—C32—H32	120.00
C2—C3—H3	120.00	S1—C33—H33A	109.00
C4—C3—H3	120.00	S1—C33—H33B	109.00
C5—C4—H4	120.00	S1—C33—H33C	109.00
C3—C4—H4	120.00	H33A—C33—H33B	109.00
C6—C5—H5	120.00	H33A—C33—H33C	109.00
C4—C5—H5	120.00	H33B—C33—H33C	109.00
C8—C7—H7	119.00	S1—C34—H34A	109.00
N1—C7—H7	119.00	S1—C34—H34B	110.00
C13—C12—H12	120.00	S1—C34—H34C	109.00
C11—C12—H12	120.00	H34A—C34—H34B	110.00
C14—C13—H13	119.00	H34A—C34—H34C	109.00
C12—C13—H13	119.00	H34B—C34—H34C	109.00
C13—C14—H14	121.00		
			
C6—N1—C7—C8	−174.14 (15)	C7—C8—C9—N2	−172.41 (15)
C7—N1—C6—C5	5.4 (2)	C10—C8—C9—O2	−177.61 (16)
C7—N1—C6—C1	−176.59 (15)	C7—C8—C10—O3	−3.3 (3)
C9—N2—C11—C12	−18.0 (2)	C7—C8—C10—N3	175.97 (15)
C11—N2—N3—C10	171.57 (13)	C7—C8—C9—O2	8.7 (3)
C11—N2—C9—O2	10.8 (3)	C9—C8—C10—O3	−177.15 (17)
N3—N2—C11—C16	0.8 (2)	C9—C8—C10—N3	2.08 (18)
C9—N2—C11—C16	163.72 (15)	N2—C11—C16—C15	177.38 (15)
C9—N2—N3—C10	5.64 (17)	C12—C11—C16—C15	−0.9 (2)
C11—N2—C9—C8	−168.25 (14)	N2—C11—C12—C13	−177.88 (14)
N3—N2—C11—C12	179.05 (14)	C16—C11—C12—C13	0.4 (2)
N3—N2—C9—O2	174.78 (15)	C11—C12—C13—C14	0.4 (2)
N3—N2—C9—C8	−4.22 (16)	C12—C13—C14—C15	−0.7 (3)
N2—N3—C10—C8	−4.60 (17)	C13—C14—C15—C16	0.1 (3)
N2—N3—C10—O3	174.69 (15)	C14—C15—C16—C11	0.7 (3)
C23—N4—C22—C17	−171.75 (16)	O4—C17—C22—C21	179.01 (16)
C22—N4—C23—C24	179.09 (16)	C18—C17—C22—N4	179.75 (16)
C23—N4—C22—C21	9.1 (3)	O4—C17—C22—N4	−0.2 (2)
N6—N5—C27—C28	174.88 (15)	O4—C17—C18—C19	−179.90 (17)
N6—N5—C27—C32	−4.1 (2)	C22—C17—C18—C19	0.2 (3)
C27—N5—C25—C24	−175.58 (15)	C18—C17—C22—C21	−1.1 (3)
N6—N5—C25—O5	174.60 (15)	C17—C18—C19—C20	0.5 (3)
C25—N5—C27—C28	−15.0 (3)	C18—C19—C20—C21	−0.4 (3)
C27—N5—C25—O5	3.8 (3)	C19—C20—C21—C22	−0.5 (3)
C25—N5—C27—C32	166.08 (16)	C20—C21—C22—C17	1.2 (3)
C27—N5—N6—C26	179.47 (14)	C20—C21—C22—N4	−179.68 (16)
C25—N5—N6—C26	7.45 (17)	N4—C23—C24—C26	1.5 (3)
N6—N5—C25—C24	−4.77 (17)	N4—C23—C24—C25	176.69 (16)
N5—N6—C26—O6	172.99 (15)	C26—C24—C25—N5	0.50 (18)
N5—N6—C26—C24	−6.87 (18)	C23—C24—C26—O6	0.2 (3)
C2—C1—C6—N1	−176.10 (14)	C23—C24—C26—N6	−179.97 (16)
O1—C1—C6—N1	4.4 (2)	C25—C24—C26—O6	−175.85 (17)
O1—C1—C6—C5	−177.50 (15)	C25—C24—C26—N6	4.01 (19)
C6—C1—C2—C3	−0.9 (2)	C23—C24—C25—O5	5.4 (3)
O1—C1—C2—C3	178.54 (16)	C23—C24—C25—N5	−175.32 (17)
C2—C1—C6—C5	2.0 (2)	C26—C24—C25—O5	−178.81 (17)
C1—C2—C3—C4	−0.7 (3)	N5—C27—C28—C29	179.85 (15)
C2—C3—C4—C5	1.2 (3)	C32—C27—C28—C29	−1.2 (3)
C3—C4—C5—C6	−0.1 (3)	N5—C27—C32—C31	179.89 (16)
C4—C5—C6—N1	176.44 (15)	C28—C27—C32—C31	0.9 (3)
C4—C5—C6—C1	−1.5 (2)	C27—C28—C29—C30	0.5 (3)
N1—C7—C8—C10	6.4 (2)	C28—C29—C30—C31	0.4 (3)
N1—C7—C8—C9	179.11 (15)	C29—C30—C31—C32	−0.7 (3)
C10—C8—C9—N2	1.32 (17)	C30—C31—C32—C27	0.0 (3)

### Hydrogen-bond geometry (Å, º)

Cg3 and Cg6 are the centroids of the C11–C16 and C27–C32 phenyl rings, respectively.

**Table:**

*D*—H···*A*	*D*—H	H···*A*	*D*···*A*	*D*—H···*A*
N1—H1···O1	0.86 (2)	2.23 (2)	2.6235 (18)	107 (2)
N1—H1···O3	0.86 (2)	2.15 (2)	2.8265 (17)	135 (2)
O1—H1*A*···O5	0.85 (2)	1.80 (2)	2.6479 (17)	174 (2)
N3—H3*A*···O2^i^	0.88 (2)	1.90 (2)	2.7740 (17)	174 (2)
N4—H4*A*···O4	0.88 (2)	2.19 (2)	2.6019 (18)	109 (2)
N4—H4*A*···O6	0.88 (2)	2.11 (2)	2.8050 (18)	136 (2)
O4—H4*B*···S1^ii^	0.85 (2)	2.85 (2)	3.5767 (14)	145 (2)
O4—H4*B*···O7^ii^	0.85 (2)	1.76 (2)	2.6061 (18)	172 (2)
N6—H6···O6^iii^	0.88 (2)	1.92 (2)	2.7831 (19)	169 (2)
C7—H7···O3^iv^	0.95	2.40	3.055 (2)	126
C12—H12···O2	0.95	2.27	2.897 (2)	122
C16—H16···N3	0.95	2.42	2.775 (2)	102
C28—H28···O5	0.95	2.27	2.893 (2)	123
C32—H32···N6	0.95	2.46	2.801 (2)	101
C34—H34*B*···O3^v^	0.98	2.43	3.403 (3)	175
C29—H29···*Cg*3^vi^	0.95	2.64	3.548 (2)	160
C33—H33*C*···*Cg*6^iv^	0.98	2.74	3.690 (2)	163

Symmetry codes: (i) *x*+1, *y*, *z*; (ii) −*x*+1, −*y*+1, −*z*+1; (iii) −*x*+2, −*y*+1, −*z*+1; (iv) *x*−1, *y*, *z*; (v) *x*−1, *y*+1, *z*; (vi) *x*+1, *y*+1, *z*.
